# Mammary Development and Breast Cancer: A Wnt Perspective

**DOI:** 10.3390/cancers8070065

**Published:** 2016-07-13

**Authors:** Qing Cissy Yu, Esther M. Verheyen, Yi Arial Zeng

**Affiliations:** 1 State Key Laboratory of Cell Biology, CAS Center for Excellence in Molecular Cell Science, Institute of Biochemistry and Cell Biology, Shanghai Institutes for Biological Sciences, Chinese Academy of Sciences, Shanghai 200031, China; cissyyu@sibcb.ac.cn; 2 Department of Molecular Biology and Biochemistry, Centre for Cell Biology, Development and Disease, Simon Fraser University, Burnaby, BC V5A 1S6, Canada; everheye@sfu.ca

**Keywords:** Wnt, mammary gland, breast cancer, development, stem cells

## Abstract

The Wnt pathway has emerged as a key signaling cascade participating in mammary organogenesis and breast oncogenesis. In this review, we will summarize the current knowledge of how the pathway regulates stem cells and normal development of the mammary gland, and discuss how its various components contribute to breast carcinoma pathology.

## 1. Introduction

The mouse mammary gland has been extensively studied as a model for breast cancer, for investigating the mechanisms underlying branching morphogenesis, as well as for studying adult stem cell behaviors orchestrated by systemic hormones and local growth factors (reviewed in [1,2,3]). It was through scrutinizing certain strains of laboratory mice that were highly susceptible to mammary tumors, in which the disease was usually transmitted from mothers to progeny through their milk, that the Mouse Mammary Tumor Virus (MMTV) was identified [4,5,6]. To identify genes that were activated due to viral insertion, MMTV mammary tumors and normal mammary tissues were compared, leading to the discovery of the first mammalian Wnt gene, *int-1*, later called *Wnt1* [7]. The identification of the *Wnt1* gene was amongst the great advances in the discovery of proto-oncogenes; it also represents the starting point of the Wnt field [8]. *Wnt1* encodes a secreted growth factor that activates a highly conserved signaling cascade. Since 1982 many Wnt family members and pathway components have been uncovered and implicated in a broad spectrum of biological events. In this review, we will summarize the recent literature on the prominent contribution of Wnt signaling to mammary development, with an emphasis on recent studies that provide insights into how Wnt signaling is integrally involved in mammary stem cell maintenance, basal cell fate determination, and basal-like type breast cancer.

## 2. Wnt Signaling Cascades

Human and most mammalian genomes harbor 19 *Wnt* genes, falling into 12 evolutionarily conserved Wnt subfamilies [9]. Wnt proteins are secreted cysteine-rich glycoproteins sharing a high degree of sequence homology. Wnt ligands initiate signal transduction through engaging a heterodimeric receptor complex consisting of a Frizzled (Fzd) family transmembrane receptor and a member of Lrp5/6 (low density lipoprotein related proteins 5 or 6) family. Wnt-Fzd interactions are promiscuous in nature, with a single Wnt capable of binding to multiple Fzd proteins and vice versa. The intracellular signaling activated by Wnt proteins is organized into two categories: canonical and non-canonical. These mechanisms have been the subject of numerous reviews [9,10,11,12] and therefore will only be described here briefly.

### 2.1. Canonical Wnt Signaling

The defining feature of activated canonical Wnt signaling is the nuclear accumulation of β-catenin, hence canonical Wnt signaling is often referred to as Wnt/β-catenin signaling. Thus far, Wnt/β-catenin signaling remains the most studied and the best understood response to Wnt ligand stimulation. In brief, in the absence of Wnt ligand binding, newly synthesized β-catenin is targeted for destruction by a complex comprising two scaffolding proteins: tumor suppressors adenomatous polyposis coli (APC) and Axin (Axin1 or Axin2); and two kinases: casein kinase 1(CK1) and glycogen synthase kinase 3β (GSK3β). β-Catenin is sequentially phosphorylated by the kinases [13], followed by ubiquitination and subsequent degradation by the 26S proteasome [14]. Upon Wnt binding to the receptor complex, phosphorylated Lrp5/6 receptor directly interacts with Axin, while Fzd binds to an Axin-binding protein, Dishevelled (Dvl), resulting in the inactivation of the destruction complex, hence inhibiting β-catenin degradation. Consequently, β-catenin accumulates in the cytoplasm and enters the nucleus where it binds a TCF (T-cell factor)/Lef family transcription factor for target gene activation [15]. The vertebrate genome encodes four highly similar TCF/Lef proteins. In the absence of β-catenin, TCF/Lef proteins repress target genes through direct association with co-repressors such as Groucho. Once β-catenin enters the nucleus, it replaces Groucho and interacts directly with TCF/Lef factors as well as co-activators such as B-cell lymphoma 9/Legless (BCL9/LGS) and Pygopus (pygo), promoting transcription of target genes [16]. The canonical Wnt cascade participates in a broad range of biological processes by regulating a large number of target genes. In the context of the mammary gland, microarray profiling has been performed in MMTV-Wnt1 mammary tissue [17] and in normal mammary cells cultured with Wnt3a [18]. Numerous Wnt target genes have also been documented in individual studies (see Table 1 for a partial list). Some of these are known to be direct targets of β-catenin/TCF binding, while others may be upregulated by indirect mechanisms. As research in this field advances, this target gene list is being continuously revised and expanded.

Various natural inhibitors (e.g., Frizzled related proteins (sFRPs), Dickkopf family proteins (DKK) and Wnt-inhibitory factor family proteins (WIF) and agonists (e.g., Norrin and R-spondins) that regulate the Wnt ligand-receptor complex and signal transduction have been identified (reviewed in [9]). The R-spondin family (Rspos) consisting of four Rspo proteins (Rspo1–4) has attracted much attention in the past decade [39]. Rspos were first reported to synergize Wnt signaling by antagonizing the Dkk1-Lrp6 interaction [40]. Later it was revealed that Rspos are actually ligands for the orphan Leucine-rich repeat-containing G-protein-coupled receptors, Lgr4, Lgr5 and Lgr6 [41,42,43,44]. Rspo/Lgr complexes act by neutralizing the negative effect of Rnf43 and Znrf3, two transmembrane E3 ubiquitin ligases that target Wnt receptors for removal from the stem cell surface, as a result canonical Wnt signaling becomes activated [45,46].

### 2.2. Non-Canonical Wnt Pathway

Non-canonical Wnt pathways do not involve β-catenin, and in some contexts, do not even involve Wnt ligands. Several non-canonical Wnt pathways have been described. One is the Wnt/Planar Cell Polarity (PCP) pathway, in which Frizzled receptors are implicated in the establishment of planar cell polarity and in the control of polarized cell migration (reviewed in [47]). In flies, but not vertebrates, these responses appear to occur independently of a Wnt ligand. The establishment of PCP is thought to involve a set of distinct downstream messengers that include Dvl and small Rho GTPases to modulate cytoskeletal factors including RhoA, and Jnk (reviewed in [48,49]). In some cases, Frizzled receptors can induce Ca^2+^ fluxes (Wnt/Ca^2+^ pathway) [50,51]. A non-canonical Wnt intracellular response utilizes the receptor tyrosine kinase (RTK) Ror2 to inhibit β-catenin/TCF signaling and activate Jnk [52]. Another pathway is activated when Wnt proteins bind to Ryk RTKs, mostly in the context of neuronal development, resulting in the activation of Src proteins (reviewed in [53]).

Of note, researchers have attempted to subdivide Wnt ligands into two groups, “canonical” or “non-canonical”. However, the intracellular response to an individual Wnt member is strongly context-specific. Wnts that have been documented to activate non-canonical Wnt pathways are also reported to fulfill dual roles during development [53]. For example, Wnt5a can engage Ror2 to inhibit the canonical Wnt signaling pathway, while paradoxically Wnt5a can also activate the canonical pathway by directly engaging Fz4 [54] and Fz5 [55]. These studies underscore that the signaling output reflects the combination of receptors that a Wnt encounters, rather than the intrinsic nature of the Wnt itself. Another case is seen with Wnt11. Typically considered to be a non-canonical Wnt, due to its function in convergent extension movements during gastrulation in amphibians [56,57,58,59], maternally contributed Wnt11 also initiates axis formation in the early *Xenopus* embryo by causing a local accumulation of β-catenin [60]. Wnt4 has also been reported to generate diverse outputs in different model systems. *zWnt4* mRNA or *mWnt4* injected into zebrafish embryos results in cyclopia and notochord malformation, implying activation of a non-canonical Wnt pathway [61]. Wnt4 has also been previously implicated in the PCP pathway in murine hematopoietic progenitor cells [62]. In contrast, canonical Wnt/β-catenin signaling can also be activated by Wnt4 in many tissues, including kidney [63,64,65], muscle [66], blood [67] and *Drosophila* mid gut [68]. In mammary cells, Wnt4 can also activate Wnt/β-catenin canonical signaling as seen by activation of a TCF-dependent luciferase reporter and *Axin2* expression, while both of the above activities can be suppressed by Dkk1 [69]. Therefore, in light of such knowledge, we suggest that it would be overly simplified and improper to refer to an individual given Wnt as participating in only a “canonical” or “non-canonical” pathway.

## 3. Wnt Signaling in Mammary Development

Wnt signaling pathways have been implicated in almost all stages of mammary development and play instrumental roles in regulating mammary stem cells (MaSCs) (reviewed in [70,71,72]). During various stages of mammary morphogenesis, the presence of numerous members of the Wnt signaling pathway has been documented, including a wide range of Wnt ligands, receptors, downstream effectors and DNA-binding proteins. Studies have been conducted in order to elucidate the Wnt signaling activities that influence mammary development.

### 3.1. Wnt Signaling in Embryonic Mammary Development

**Figure 1:**
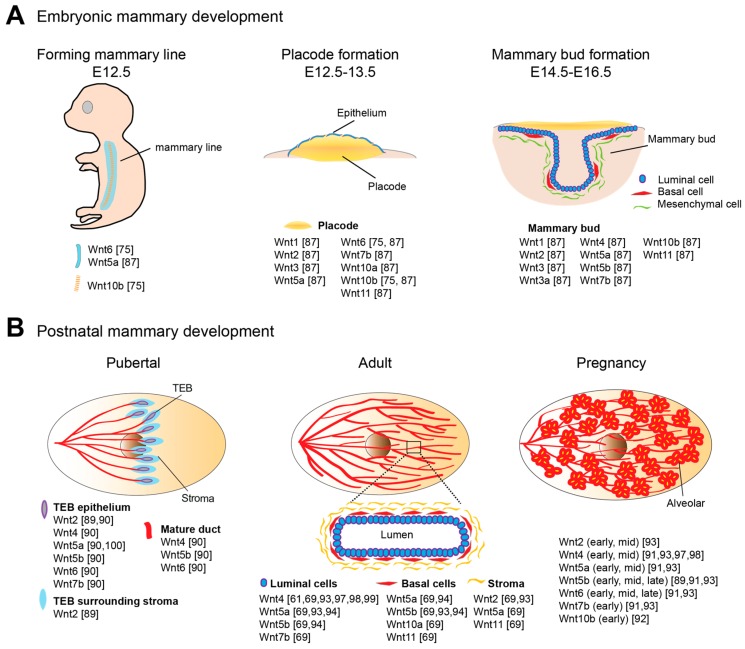
The expression of Wnts at various stages of mammary development. Expression pattern of Wnt members documented at key morphogenesis stages, including mammary line, placodes, and mammary bud formation during embryogenesis (**A**); and puberty, adult and pregnancy stages during postnatal development (**B**).

Wnt signaling is pivotal for specification and morphogenesis of the mammary gland [71,73]. Murine mammary morphogenesis initiates on embryonic day 10.5 (E10.5), characterized by invaginations of the epidermis (Figure 1A). Canonical Wnt signaling activities define the cells that will form mammary lines and later becomes restricted to the cells forming placodes. By E12, two mammary lines have formed, each giving rise longitudinally to five distinctive mammary placodes [74]. Wnt10b expression is detected within the forming mammary lines and represents the earliest discernible event in murine embryonic mammary gland development [75]. In addition, Wnt6 is initially expressed in the surface ectoderm as a broader band surrounding the Wnt10b-expressing mammary lines. Both Wnt10b and Wnt6 expression patterns are refined to placodes 1.5 days later [75]. Cultured embryos supplemented with Wnt3a or lithium chloride (LiCl), a potent Wnt activator, exhibited the formation of efficient placode-like structures with augmented Wnt10b expression, suggesting that canonical Wnt signaling regulates placode development. Interfering with positive regulators of the Wnt pathway, e.g., Lrp6, Lrp5, Lef1 and Pygo2, results in placodal impairments, ranging from loss to reduced size and degeneration. In contrast, stimulating β-catenin signaling leads to the accelerated induction of placodes and placodal markers (Wnt10b and T-box transcription factor-3) [76,77,78,79]. Embryos of genetically modified mouse models, in which either Dkk1 is overexpressed or Lrp6 or Lef1 is deficient, have been reported to have missing placodes [78,79,80]. Lef1 appears to be a key Wnt component required for early mammary morphogenesis. Lef1 protein is first expressed in the mammary epithelial cells at E11/12. Later at E14/15, Lef1 expression is found to be restricted to mesenchyme surrounding each bud [81]. 

This induction of altered expression between cell lineages has been shown to be a result of paracrine communication from the mammary epithelium to surrounding mesenchyme, mediated by parathyroid hormone-related peptide (PTHrP) [81]. The induction of Lef1 in mesenchyme is crucial for the maintenance of mammary epithelial fate and further ductal morphogenesis at E16, indicating precise temporally controlled Wnt signaling. The next stage of mammary morphogenesis occurs after placodes invaginate to form mammary buds, and then subsequently primary sprouts, a process also dependent on canonical Wnt signaling. Bud invagination is dependent upon mammary mesenchyme specification, while activation of Wnt/β-catenin signaling in mesenchyme is dependent on PTHrP [81,82,83,84,85,86]. While recombinant Wnt3a accelerates the placode invagination process, placodes from Lef1^−/−^ mice fails to invaginate further [19,79,87]. In both Pygo2^−/−^ mice and Lrp6^−/−^ mice, the processes of sprouting into the mesenchyme and branching are impaired [76,77]. Once sprouting is completed, a primitive ductal tree has formed.

### 3.2. Wnt Signaling in Postnatal Mammary Development

The majority of mammary development occurs postnatally in several dynamic processes. At the onset of puberty at around 3 weeks of age in mice, in response to ovarian hormones, the preexisting rudimentary ductal tree rapidly expands and extends across the fat pad, occupying the whole mammary fat pad by approximately 7 weeks of age (Figure 1B). Terminal end buds (TEBs) are highly proliferative, club-shaped structures formed at the tips of growing ducts for penetrating the fat pad, particularly during puberty. The outer cell layer of the TEB, also called cap cells, differentiates into basal cells, together with the inner-layer luminal cells forming the bilayered ductal tube. Highly elongated basal cells and cuboidal luminal cells compose the two main cellular lineages of the nulliparous and non-pregnant mammary gland. The primary ductal structure is generated by TEB bifurcation. Secondary branches sprout laterally from the primary ducts, forming a tree-like pattern of ductal networks. Short tertiary branches forms during each estrous cycle under cyclical ovarian hormone stimulation, while extensive structural remodeling and full maturation of the alveolar buds into units capable of milk secretion occur during pregnancy and lactation. The process of involution is initiated post weaning, whereby the gland is remodeled back to its pre-pregnancy state (reviewed in [88]).

Both canonical and non-canonical signaling play important roles in the robust pubertal ductal growth phase and in adult mammary epithelium maintenance. Several members of the Wnt ligand family are expressed in the pubertal mammary gland, with a few Wnts showing peaks of expression during adolescence, including Wnt5a and Wnt7b, which are particularly enriched in the highly proliferative TEB structures, and Wnt2 in surrounding stroma (Figure 1) [89,90]. The expression of multiple Wnt members is detected in adult mammary tissues as well as a range of Fzd receptors [91,92,93]. In addition, secreted inhibitors of Wnt signaling, such as sFRP family, WIF1 and Dkk1 may also be functionally involved (reviewed in [1]). Recent studies using isolated basal and luminal cells to profile Wnts and Wnt partners have offered information at a higher level of resolution [69,94]. In the adult mammary gland, Wnt2, Wnt5a and Wnt11 are the highest expressed Wnts in stromal cells; Wnt5a, Wnt5b, Wnt10a, Wnt11 and the antagonists, Dkk3 and Sfrp1, are detected in basal cells; Wnt4, Wnt5a, Wnt5b, Wnt7b and the agonist, Rspo1 are expressed in luminal cells [69].

More direct evidence for the significance of Wnt signaling has emerged from studies using transgenic mouse models, with the earliest being the *MMTV-Wnt1* model, where accelerated ductal outgrowth was observed as early as 1–2 weeks of age [95]. However, the principal phenotype of ectopic Wnt1 and Wnt10b expression in transgenic strains is lobuloalveolar hyperplasia rather than ductal hyper-growth [92,95,96], highlighting a need for investigation into the action of other endogenous Wnts in the mammary gland. Ectopic side-branches were observed in transplantation assays upon Wnt4 overexpression [97]. On the other hand, *MMTV-Wnt4* transgenic mice display no phenotype [61]. Studies using a Wnt4 loss of function strategy yield more consistent results. Early pregnancy side-branching defects in Wnt4 null (*Wnt4^−/−^*) transplants have been reported [98]. A recent study further demonstrated the significance of Wnt4 in mammary development. The compromised regenerative ability of a Wnt4 null epithelium becomes apparent in the 3rd passage of in vivo serial transplantation [99]. Another study reports the synergistic action of Wnt4 and Rspo1, as knockdown of Wnt4 and Rspo1 expression can completely abolish the regenerative capability of the mammary cells in primary transplantation [69]. Canonical Wnt signaling has been implicated as the key signaling pathway for basal cell fate determination and mammary stem cell self-renewal, a topic on which we will elaborate in the next section.

Non-canonical Wnt signaling has been shown to participate in the negative regulation of mammary epithelial outgrowths, adding another level of complexity. Mice with disrupted expression of Wnt5a (*Wnt5a^−/−^*) displayed precocious and extended developmental capacity with larger terminal end buds (TEBs), rapid ductal elongation and increased branching frequency, hence suggesting the inhibitory role of Wnt5a during normal mammary development is necessary to ensure proper branching morphogenesis [100]. TGFβ has been identified as the upstream factor that induces Wnt5a expression in the mammary gland [100]. A recent study found Ror2 as the receptor for mediating Wnt5 (Wnt5a and Wnt5b) induced non-canonical Wnt signal transduction in mammary cells [94]. Ror2 is predominantly expressed in both basal and luminal cells. Deletion of Ror2 results in increased branching at the expense of ductal elongation [94]. The mechanism of Wnt5 induced mammary growth inhibition has also been linked to an extracellular proteinase effector matrix metalloproteinase 3 (MMP3). Through a yeast two-hybrid screen, specific interactions between MMP3 and both Wnt5 members were identified. MMP3 inhibits the ligand activity of Wnt5a and Wnt5b by sequestration and proteolytic cleavage of Wnt5’s C-terminal domain. MMP3 is predominantly expressed in stromal cells, while both Wnt5a and Wnt5b are mainly expressed in luminal cells. It is proposed that Wnt5 and MMP3 both function in a paracrine manner, targeting cells in the basal layer, which harbors mammary stem cells (MaSCs). MMP3 induces hypermorphic epithelial growth and activation of canonical Wnt signaling. Thus MMP3 might skew the balance between canonical and non-canonical Wnt signaling in favor of the promotion of growth and tumor formation [101].

### 3.3. Wnt/β-Catenin Signaling in MaSC Regulation and Basal Cell Fate Determination

MaSCs are the key drivers of mammary gland self-renewal and differentiation throughout development, not only in active growth phases, but also for the maintenance of tissue homeostasis [102]. The existence of MaSCs was demonstrated many decades ago by reconstitution of the mammary gland after transplantation of a mammary epithelial fragment into a cleared fat pad [103]. Recent advances have identified functional MaSC-enriched populations using selection based on cell surface marker expression, followed by mammary reconstitution assays, laying the groundwork for establishing the cellular hierarchy of the mammary epithelium. These surface markers for mouse basal cells are CD24^+^, CD29^hi^/CD49^hi^ [104,105] and the human counterparts are CD49f^hi^, EpCAM^−^ [106,107]. A recent study suggests that a further enriched MaSC group within the basal population can be identified by utilizing an additional surface marker Protein C Receptor (Procr) [18]. The use of surface markers for isolation and enrichment of stem cells has facilitated the investigation of the molecular signaling pathways regulating MaSC self-renewal and lineage commitment. A growing body of evidence indicates that Wnt is a niche factor and Wnt signaling is a key regulator of MaSC activities [18,21,22,69,108,109]. It should be noted that the terms “MaSCs” and “MaSC-enriched basal cells” have appeared interchangeably in many studies in the past decade, when describing cells capable of in vitro colony formation or repopulation after transplantation, or both. Indeed, recent lineage tracing studies have reinforced that basal cell populations harbor bipotent stem cells able to give rise to basal and luminal progeny [18,110]. Some confusion arises from the term “basal stem cell”, referring to unipotent stem cells which contribute predominantly to basal cells in lineage tracing, yet they are capable of generating both basal and luminal cells upon transplantation [108,111,112,113,114]. In parallel, the unipotent stem cells residing in the luminal layer are also referred to as luminal progenitors [110]. In light of the distinct contributions revealed by lineage tracing studies, it is clear that “basal stem cell” is not equivalent to “MaSC”, and probably “basal stem/progenitor cell” is a more accurate term to reflect the cells that contribute only to basal cells. For the purpose of this review, we will use “basal cells” if the subject has only been analyzed by surface marker expression (CD24^+^, CD29^hi^/CD49^hi^); we will use “MaSCs” or “MaSC-enriched basal cells” dependent on the context, if the subject has been examined by ex vivo and in vivo functional assays.

Studies have directly addressed Wnts as niche factors for MaSCs [22,69]. In 3D Matrigel cultures, addition of Wnt3a protein to basal cell primary cultures results in a 7-fold increase in the number of secondary colonies. Cells cultured with Wnt3a showed continued expansion while going through multiple passages, producing increasing number of colonies. Moreover, to test the stem cell capabilities of the in vitro cultured cells, such colonies were transplanted in vivo, revealing that they retained the ability to reconstitute mammary glands efficiently [22]. It is important to note that, despite its potent effects in vitro, Wnt3A is not expressed in the mammary gland [93]. Wnt4 and Rspo1 secreted from luminal cells appear to represent the in vivo Wnt signal regulating MaSCs. Expansion of basal colony numbers is observed when co-culturing with luminal cells overexpressing Wnt4, reminiscent of the Wnt3a effect. In contrast, overexpression of Wnt7b, another Wnt member displaying a luminal expression pattern, cannot recapitulate the colony expansion phenomenon [69]. Hence MaSCs within the basal cell cultures can be directly targeted by certain Wnt proteins and Wnt signaling is instrumental for MaSC self-renewal and expansion activities.

In an attempt to identify Wnt targets specifically expressed in MaSCs, microarray analysis of cultured MaSC-enriched basal cells was performed, leading to the discovery of Procr, a surface marker that can be used to further enrich for MaSCs [18]. Of note, known Wnt targets, such as Axin2 also exhibit elevated expression in basal cells cultured with Wnt3a [18]. Procr^+^ basal cells exhibit 5-fold higher colony formation efficiency in primary cell culture and about 6-fold higher repopulating frequency in transplantation when compared to total basal cells. Studies using a *Procr-tdTomato* knock-in allele demonstrate that Procr^+^ MaSCs can reside in both duct or tip regions in perinatal or newborn mammary glands, however Procr is excluded from the TEB, a highly proliferative structure at the growing tips of the ducts during puberty [18]. Interestingly, the two Wnt-responsive populations, Procr^+^ and Axin2^+^ cells, are in two distinct compartments during puberty. TEBs represent active zones for Wnt/β-catenin activity with robust *Axin2* expression [94,108]. In post-puberty mature mammary glands, after the TEB structures have vanished, Axin2 and Procr still mark different basal subpopulations [18]. The factors that specify the distinction between the two Wnt-responsive populations during mammary development remain to be determined. It is very likely that Procr^+^ MaSCs receive other niche cues in addition to Wnt signals, which would jointly define the identity of these MaSCs [115]. It is not surprising that a hierarchy exists within the Wnt-responsive mammary cells, considering that they vary in repopulating efficiencies and in lineage commitments [18,22,108]. A comparison of their robustness in stem cell assays will be further discussed in the next section together with results of lineage tracing experiments.

Based on the lineage hierarchy that has been delineated in other tissues, below the multipotent stem cells exists a population of cycling stem/progenitor cells (transient amplifying cells) (reviewed in [116]). It has been proposed that the basal cells of the TEB (cap cells) represent a MaSC reservoir that drives the formation of more mature epithelial lineages during ductal extension and primary branch formation [117,118]. Given their repopulating ability, their highly proliferative nature, and their robustness in response to Wnt signals, cap cells are reminiscent of cycling stem/progenitor cells. A recent report demonstrates that Wnt signaling in dividing cap cells is elevated by a mechanism dependent on Cyclin Y (Ccny) and Cyclin Y-like 1 (Ccnyl1) [119]. During mitosis, Ccny localized at the plasma membrane recruits Cyclin-dependent kinase 14 (Cdk14) for the phosphorylation of Lrp6 on PPPSP motifs, which sensitizes Lrp6 for upcoming Wnt signals [120,121]. Ccny/Ccnyl1 expression and phosphorylated Lrp6 levels are elevated in mitotic mammary cells. In particular, *Ccnyl1* expression is enriched in cap cells, colocalizing with *Axin2* expression. Deletion of Ccnys diminishes the regenerative capability of MaSC-enriched basal cells in transplantation and lineage tracing experiments. Hence, the enhancement of Wnt signaling in mitosis is essential for the stem/progenitor cell property maintenance during division [119]. This study uncovers a cell cycle and Wnt signaling feed forward mechanism in cycling stem/progenitor cells for their expansion, shedding new light on the molecular mechanisms orchestrating cell cycle progression and maintenance of stem cell properties. In addition to *Axin2* and *Ccnyl1*, *s-ship* has also been reported to be expressed in cap cells of TEBs [122]. The number of s-Ship-expressing cells expands in *MMTV-Wnt1* tumors but not in *MMTV-ErbB2* tumors, suggesting that this population is regulated by Wnt signaling. s-Ship-expressing cells were seen in mammary buds at embryonic development, TEBs at puberty and alveolar buds in pregnancy mammary glands visualized by a transgenic GFP reporter inserted after the s-SHIP promoter. In contrast, expression was not observed in mature mammary glands or during other developmental stages such as lactation, involution and post-involution, in which MaSCs are relatively quiescent. s-Ship-expressing cap cells possess regenerative capacity as demonstrated by serial transplantation experiments. These observations suggest that s-Ship-expressing cells are enriched for active MaSCs [122].

In line with the notion that Wnt/β-catenin signaling promotes MaSC self-renewal and expansion, it has been reported that attenuating Wnt/β-catenin signaling limits MaSC self-renewal. Slit2, secreted from luminal and basal cells, binds to the Robo1 receptor, whose expression is restricted to basal cells and is upregulated by TGFβ [123,124]. It is noteworthy that TGFβ induces Wnt5a expression in luminal cells [100], while inducing Robo1 expression in basal cells [123]. Slit/Robo1 signaling inhibits Wnt/β-catenin signaling through increasing the cytoplasmic and membrane pools of β-catenin at the expense of its nuclear pool, thus limiting basal cell proliferation [123]. Interestingly, loss of either Robo1 or Robo2 similarly increases nuclear β-catenin levels in mammary basal cells, but with very different outcomes: loss of Robo1 leads to increased basal cell proliferation [123], whereas loss of Robo2 results in increased MaSC self-renewal, as evidenced by an increase in the number of generations that MaSCs can be serially transplanted [125]. It was further shown that Slit/Robo2 counteracts the Wnt-mediated repression of p16^INK4a^ expression, as a result promoting MaSC senescence and impairing self-renewal [125]. These studies support the notion that Wnt/β-catenin signaling is involved in the dynamics of distinct basal cell subpopulations, one which is likely MaSC and another which could be a more differentiated basal subpopulation.

In general, Wnt/β-catenin signaling plays critical roles in the determination of mammary basal cell fate [21,25,126], with the exception that Wnt-responsive Axin2^+^ cells in embryonic stages are implicated in luminal lineage specification (see next section). The Notch signaling pathway dictates luminal lineage determination [127,128,129]. Coordinated efforts of multiple epithelial cell lineages are essential for establishing an organized bilayered ductal network. The significance of the Wnt and Notch balance is best illustrated by a recent finding that the histone methylation reader Pygo2 is necessary for suppressing luminal and alveolar differentiation of the MaSC/basal population by coordinating the activity of the Wnt and Notch pathways [126]. Pygo2, previously known as context-dependent transcriptional Wnt/β-catenin signaling co-activator, is required for β-catenin association with both *Axin2* and *Notch3* gene regulatory regions. Pygo2 within the MaSC/basal cells engages β-catenin to bind to the *Notch3* locus, retaining Notch3 in a “bivalent” yet repressed chromatin structure, thereby tilting the Wnt and Notch signaling balance towards promoting a basal-favorable status by actively suppressing luminal differentiation [126]. In the absence of Pygo2, the MaSC/basal cells adopts a more luminal-like cell phenotype, further reinforcing the role of Pygo2/β-catenin in ensuring basal cell identity.

A recent report indicates that ΔNp63, the ΔN isoform of p63, plays a prominent role in basal cell fate determination through modulation of Fzd7 expression and Wnt signaling activities [21]. ΔNp63 is predominantly expressed in basal cells. Suppressing ΔNp63 expression results in diminished MaSC repopulating ability and delays in mammary development. Strikingly, overexpression of ΔNp63 enables luminal cells to generate outgrowths in transplantation assays, suggesting that ΔNp63 can induce luminal cells to acquire MaSC properties. Fzd7, identified as a direct target of ΔNp63, is the major downstream effector of ΔNp63 in basal cells. Overexpression of Fzd7 in ΔNp63-deficient mammary cells leads to partial rescue of the loss of MaSC phenotypes in culture and in repopulation assays [21]. The significance of ΔNp63 in basal cell fate determination has also been documented in a recent study associated with LBH (limb bud and head) [25]. The expression of LBH, a target of Wnt signaling, is restricted to basal and stromal cell populations. Inactivation of LBH results in delays in mammary development and increased luminal differentiation at the expense of basal lineage specification. It is proposed that LBH induces ΔNp63 expression to promote a MaSC/basal fate and repress luminal differentiation [25]. The above studies summarize recent advance in understanding the mechanisms though which Wnt/β-catenin signaling governs basal cell fate decision.

### 3.4. Lineage Tracing Employing Wnt-Targets

The approach of using transplantation and ex vivo assays to examine the cell fate of freshly dissociated mammary epithelial cells enables quantification of MaSC regenerative capacities. It has been suggested that such transplantation assays may not reflect the true behavior of stem cells, as the stress induced by the process of tissue dissociation and transplantation into a cleared fat pad may result in cell plasticity, and impart properties that they would not normally exhibit in intact tissues (reviewed in [129]). Recent lineage tracing studies have bypassed such tissue manipulation and have revolutionized our knowledge of Wnt-responsive cell behaviors during mammary development [18,108,110,111,113].

The regenerative capacity of Axin2^+^ mammary cells has been demonstrated in transplantation experiments [22]. To assess the contribution of Wnt-responsive (Axin2^+^) cells in development, van Amerongen and colleagues have used an *Axin2^CreERT2^* knockin mouse model to label cells throughout mammary gland development. Labeling Axin2^+^ cells in the embryo predominantly yielded cells of the luminal lineage in the adult mammary gland, whereas cells labeled in prepubescent (2-week-old pups) resulted in tracing of only basal cells. These results highlight the cell fate switching of Axin2^+^ cells, in another words, Wnt/β-catenin signal activation in distinct cells at different stage of mammary development. Indeed, in puberty, *Axin2* expression is detected in both basal and luminal cells of TEBs. Tracing of Axin2^+^ cells at this stage resulted in labeling of both basal and luminal cells in adults. When tracing in nulliparous adults, a stage when *Axin2* expression remains restricted mainly to the basal layer, labeling is seen predominantly in basal cells. Although not observed through immunostaining, it is noteworthy that a small fraction (1.5%) of labeled cells are detected in the luminal cell compartment by FACS analysis. Interestingly, these rare potentially bipotent events can be better visualized when the traced animal is pregnant, during which labeled luminal cells are detected adjacent to labeled basal cells, implying that these labeled cells originated from a bipotent MaSC [108].

Lgr5, a recognized Wnt target and stem cell marker in the intestine [130], also exhibits an expression pattern switch during mammary development. In newborns, Lgr5 expression is restricted to the luminal layer and lineage tracing experiments using the Lgr5*^CreERT2^* knock in mouse model indicate the luminal fate of the progeny in adult mammary glands. However, in prepubescent animals, the expression of Lgr5 is switched to the basal compartment, and labeling initiated at postnatal day 12 reveals only basal cells when analyzed in adults [111]. It is unclear whether Lgr5 is a Wnt/β-catenin target in the mammary gland based on some recent lines of evidence (reviewed in [129]). First, Lgr5^+^ cells were not enriched for expression of the Wnt pathway target gene *Axin2* [112]. Second, in microarray analysis of cultured MaSC-enriched basal cells, Lgr5 was not detected among the candidates including Axin2 and Procr, whose expressions are upregulated in the presence of Wnt3a proteins [18]. In contrast, a recent study reported the upregulation of Lgr5 expression in *MMTV-Wnt1* mammary tumor cells and in human breast cancer cell lines when incubated with Wnt3a [20]. Nevertheless, despite the uncertainty of the upstream regulation of Lgr5 expression in mammary cells, it is intriguing that Lgr5 and Axin2 exhibit a similar expression pattern switch from luminal- to basal-specific in prepubescent animals, suggesting a common regulatory mechanism in these two populations.

Recent work by Wang et al. identified Procr as a novel Wnt target in mammary cells [18]. Procr expression is detected in both mesenchymal and basal cells, yet excluded from luminal cells throughout mammary development. The percentage of Procr^+^ basal cells remains stable, consisting of 3%–8% of basal cells (depending on the genetic background) during various developmental stages. Lineage-tracing studies using *Procr^CreERT2^* knock in mice revealed that Procr^+^ cells contribute to both basal and luminal lineages at all developmental stages. Ablation of Procr^+^ cells during puberty using diphtheria toxin fragment A (DTA) resulted in delayed epithelial extension reflecting their essential role in mammary development [18]. This study identified Procr^+^ basal cells as multipotent MaSCs. Moreover, Procr^+^ MaSCs exhibit partial EMT characteristics and have 2-fold lower expression of basal keratins (K14 and K5), as well as 3-fold lower expression of smooth muscle related factors (Acta2 and Myh11) compared to the rest of the basal cell population [18,131]. These differences in marker expression may explain the inefficiency of tracking these multipotent MaSCs [113,114] and the conflicting results obtained [110,113] when using keratin-based or SMA-based lineage tracing.

Some special effort has been put towards addressing the relationship between different basal subpopulations. FACS analysis using an Lgr5-EGFP mouse in combination with a Procr antibody demonstrated that Lgr5^+^ and Procr^+^ basal cells are distinct populations [18]. RNAseq and qPCR analysis on isolated Procr^+^ and Procr^−^ basal cells confirmed that Lgr5 is expressed in the Procr^−^ cells [18]. Of relevance, Procr^+^ MaSCs demonstrate the highest repopulation efficiency, and Lgr5^+^ basal cells repopulate at a ten-fold lower efficacy [18]. This finding about Lgr5 is different from a previous report [112], yet is consistent with two other studies [110,111]. Importantly, Procr^−^, Lgr5^−^ cells failed to repopulate in vitro or in vivo, suggesting MaSCs are depleted in this population [18].

In parallel, experiments have also been carried out to compare the Procr^+^ and Axin2^+^ populations. Procr^+^ cells are not enriched for the expression of *Axin2* as shown by RNAseq and qPCR analyses, suggesting these two populations are largely not overlapping [18]. To further characterize the extent of overlap, Procr^+^ and Procr^−^ basal cells were FACS-isolated from *Axin2^lacZ/+^* 8-week old nulliparous mammary glands for X-gal staining. A very small percentage of Procr^+^ cells (about 0.045%–0.12% of basal cells, given that Procr^+^ cells are about 3%–8% of basal cells) were found to express *Axin2^lacZ^* [18]. This may explain that the rare bipotent events observed in lineage tracing experiments using *Axin2^CreER^* mice were likely Procr-expressing Axin2^+^ cells [108].

These lineage tracing and transplantation studies underscore the hierarchy existing within basal populations. Specifically, transplantation experiments indicate that there are multiple, molecularly distinct, basal cell subpopulations that are capable of repopulation, though the robustness varies. Lineage tracing studies suggest that Wnt/β-cat signaling is involved in both multipotent MaSC and lineage committed stem/progenitor dynamics, highlighting that lineage tracing is a very powerful technique to interrogate cell fate in its native habitat.

### 3.5. Hormones Acting through Local Wnt Factors Regulate MaSC Behavior

The concerted action of systemic hormones and local growth factors tightly and precisely regulate mammary gland development and function throughout reproductive life. Estrogen and growth hormones drive ductal elongation at the onset of puberty, whereas oscillations of estrogen and progesterone sustain the ductal network remodeling during each estrus cycle. Progesterone and prolactin induce the formation of alveolar structures capable of milk production during pregnancy (Reviewed in [132,133,134,135]). The natural estrous cycle in mice can be divided into four stages, proestrus, estrus, metoestrus and dioestrus. Proestrus and estrus in rodents are comparable to the follicular phase of the human menstrual cycle, featuring increased estrogen levels. Metoestrus and dioestrus in mice are similar to the luteal phase in humans, which is characterized by elevated levels of progesterone [136,137,138]. The receptors for estrogen and progesterone, ER and PR respectively, are expressed in between 30% and 50% of the luminal cells, and basal cell populations in which MaSCs reside lack both ER and PR (reviewed in [2]). Hormones non-autonomously govern the behavior of MaSCs through local Wnt factors. Recent research has put Wnt4 in the spotlight as a mediator of hormonal actions on MaSCs [69,99].

The expression of Wnt4 is regulated by both estrogen and progesterone. *Wnt4* mRNA expression is induced by progesterone and its level peaks at mid pregnancy in mice (day 10.5) [69,98]. Estrogen is involved in Wnt4 protein regulation, inducing elevated Wnt4 protein levels in vitro, and allowing Wnt4 proteins to continue to accumulate until the beginning of late pregnancy (day 15.5) [69]. The population of MaSC-enriched basal cells expands in number during pregnancy and during each dioestrus [139,140]. In nulliparous mammary glands, Wnt4 levels are correlated with the pace of basal cell expansion, exhibiting the highest expression in the dioestrus phase [69]. In situ hybridization reveled that *Wnt4* is expressed in PR^+^ luminal cells. The ability of Wnt4 to regulate MaSCs was demonstrated by in vitro culture and transplantation experiments, in which Wnt4 protein could maintain the regenerative capacity of cultured basal cells when assessed with transplantation. Likely due to the redundancy of Wnts in the mammary gland, Wnt4 deficiency resulted in rather normal mammary outgrowths in virgins and during pregnancy, either by transplantation using *Wnt4* null mammary epithelium [98], or using mammary cells with Wnt4 knocked down by shRNA in transplantation assays [69]. It was through serial transplantation, an assay more challenging to the long-term regenerative capacity of MaSCs, that the significance of Wnt4 deletion was revealed. While wildtype epithelial cells can be serially transplanted for up to seven cycles [141], *Wnt4* null epithelium exhibits reduced regenerative abilities, occupying only 10% of the fat pad by the 3rd transplant [99].

Rspo1 is another critical Wnt signaling component in MaSCs that is regulated by hormones. In situ hybridization revealed that *Rspo1* is expressed in PR- luminal cells. Rspo1 levels are indirectly influenced by estrogen through a paracrine factor emanated from hormone receptor-positive luminal cells [142]. Rspo1 exhibits robust expression during pregnancy and diestrus phase, perfectly synchronized with Wnt4 levels [69]. Rspo1 can amplify Wnt4 activities in mammary cell culture, as revealed through TCF-reporter activation and clonogenicity of basal cells. Interestingly, although Rspo1 is positioned as a helper of Wnt4, Rspo1 deficiency resulted in a stronger mammary phenotype compared to deletion of Wnt4, as evidenced by fewer side-branches in virgin animal, alveologenesis defects during pregnancy, and reduced numbers of stem cells as seen by a decreased repopulating frequency [69,143]. Wnt4 and Rspo1 double knockdown led to a failure to generate mammary outgrowths in primary transplants, demonstrating the synergistic effort of Wnt4 and Rspo1 in regulating MaSCs [69]. Interestingly, elevated Rspo1 and Wnt4 also occur during the estrus phase and late pregnancy when estrogen levels surge, suggesting additional physiological roles of Rspo1 and Wnt4, e.g., regulation of luminal cell proliferation. Indeed, recent studies have reported the effect of Wnt4 and Rspo1 on luminal cells [140,144].

RANKL (receptor activator of NF-kB ligand) is another downstream target of progesterone that has been proposed to be a hormone-mediated local factor for MaSCs [139]. However, serial transplantation experiments in a recent study using *RANKL*^+/+^ and *RANKL*^−/−^ mammary epithelium demonstrated equal regenerative potential, indicating that RANKL is dispensable in MaSC regulation [99]. This is supported by MaSC culture experiments using recombinant RANKL, in which MaSC properties could not be maintained and the cultured cells lost their reconstitution abilities when transplanted [69]. The current consensus is that the RANKL pathway is essential in mammary alveologenesis [145,146]. A recent study has connected the RANKL pathway with Wnt signaling activities at this stage in mammary basal cells and luminal progenitors [144]. Deletion of the RANK receptor in *K5-Cre*; *Rank^fl/fl^* mammary cells resulted in loss of progesterone-induced alveolar differentiation, accompanied by a reduction of *Axin2* and *Rspo1* expression. Injection of Rspo1 proteins could rescue alveolar development and reactivate Wnt/β-catenin signaling (*Axin2* expression) in these animals. Therefore, it is proposed that RANKL/RANK signaling is one of the upstream pathways to induce Rspo1 expression, thus activating Wnt/β-catenin signaling during alveogenesis [144].

### 3.6. In Vitro Culture of Mascs using Wnts and Hormones

Steered by the prominent role of Wnt signaling in stem cell maintenance, Sato et al. employed Rspo1 to augment Wnt signaling and successfully expanded Lgr5^+^ intestinal stem cells in culture [147]. Subsequently, expansion of Lgr5^+^ adult stem cells of stomach and liver tissue was also accomplished by using Rspo1 [148,149]. MaSCs can be expanded in vitro under serum-free and feeder-free defined conditions when supplemented with Wnt3a [22]. A subsequent study demonstrated that Wnt4, the endogenous Wnt regulating MaSCs in vivo, can also expand MaSCs in culture [69]. However, the purification of Wnts has proven to be difficult and the availability of purified and biologically active Wnt proteins is limited. Although Rspo1 synergizes with Wnt4 in vivo in regulating MaSCs, given the rapid decrease of endogenous Wnt4 expression in mammary cell culture [69], Rspo1 is predicted to have a limited application in creating a Wnt-augmented condition as it does for intestine, stomach and liver stem cell culture [147,148,149,150]. Taking advantage of the fact that both Wnt4 and Rspo1 are under hormonal regulation, estrogen and progesterone can be engaged to recapitulate Wnt-induced MaSC expansion in vitro. In a basal and luminal cell co-culture system, hormones were applied to stimulate the endogenous expression of Wnt4 and Rspo1 in luminal cells, and MaSCs were successfully expand in culture, notably maintaining the full development potential when examined in transplantation assays [69]. This approach accurately recapitulates the in vivo physiological situation in vitro.

## 4. Wnt Signaling and Breast Cancer

Studies in mice strongly suggest that dysregulation of Wnt signaling increases breast cancer risk. Starting from the discovery of the oncogenic role of Wnt1 in murine MMTV-Wnt1 mammary tumors [7,96], a growing body of evidence has implicated Wnt/β-catenin signaling in human breast tumorigenesis. Wnt10b transgenic expression was shown to produce similar effects as MMTV-Wnt1 [92]. In addition, overexpression of Lrp6 or stabilized β-catenin, or loss of APC, resulted in mouse mammary hyperplasia or tumors (reviewed in [71]). Wnt agonist Rspo proteins have been implicated in promoting mammary tumor formation. In MMTV insertional mutagenesis screening studies involved in mammary tumorigenesis, activation of the *Rspo2* and *Rspo3* genes has been observed [151,152]. In addition, overexpression of Wnt1 and Rspo2 in mammary epithelial cells, individually or together, leads to mammary tumor formation and cells exhibit a strong EMT phenotype and are highly metastatic to lung and spleen [153]. It is noteworthy that the murine MMTV-Wnt1 model of mammary cancer shares transcriptional patterns with, and exhibits hallmarks of, human triple-negative breast cancer (TNBC)/basal-like breast cancers [154]. Furthermore several studies have reinforced the link of Lrp6 and nuclear β-catenin with TNBC [34,155,156].

Although Wnt pathway mutations are rarely found in human breast cancer, hyperactive signaling is apparent, particularly in TNBC or basal-like types (reviewed in [157]). TNBCs are clinically defined by the lack of expression of estrogen receptor (ER), progesterone receptor (PR), and the absence of amplification or overexpression of HER2 [158]. This subtype accounts for 15% to 20% of newly diagnosed breast cancer cases, is associated with a higher grade, stem cell-like characteristics, aggressive behavior, distinct patterns of metastasis, and poor patient survival [158,159,160]. Wnt/β-catenin signal activation, as defined by nuclear β-catenin and overexpression of the Wnt target cyclin D1, was preferentially found in the invasive TNBC subgroup [156], in association with poor prognosis [161,162]. Moreover, DKK1, a direct downstream target of the Wnt/β-catenin pathway [163,164,165], was found overexpressed in TNBC breast cancer as a consequence of the hyperactivation of Wnt/β-catenin signaling, also in association with poor outcome [166,167,168]. It is noteworthy that the terminology relating to TNBC and basal-like cancers tends to be used interchangeably, although they are not entirely synonymous. The first definition of basal breast cancer came from a genomics study in 2000, in which five molecular subtypes of breast cancer were recognized based on their gene expression patterns [169,170]. Genomically defined basal tumors and clinically defined TNBCs are largely overlapping types, with up to 20% of basal-like cancers expressing ER/PR or overexpressing HER2 [158,171].

Genetic mutations of APC or the β-catenin encoding gene *CTNNB1*, which are the major causal factors for hepatic and colorectal cancers [9], are not typically associated with breast cancer. Approximately 6% of breast tumors contain mutations in the APC gene and thus far there are no reports indicating CTNNB1 mutation in cases of breast cancer [172,173,174,175,176,177,178]. In contrast to the absence of molecular lesions, downregulation or epigenetic silencing of these genes occurs in up to 70% of human breast cancer cell lines and cancers [173,179,180,181,182,183,184,185,186,187,188,189].

The epigenetic inactivation of extracellular antagonists of the Wnt pathway occurs frequently and such events have been recognized as strong prognostic markers of poor outcome in breast cancers. Methylation of *DKK1* and *DKK3* promoters has been reported in breast cancers [31,190]. More frequently, loss of an extracellular antagonist by epigenetic silencing of the genes encoding sFRP family members (in particular sFRP1, sFRP2 and sFRP5) by DNA methylation has also been observed in breast cancer and is associated with an unfavorable prognosis [31,191,192]. Ectopically expressed sFRP1, which binds to and effectively competes with the FZD receptor for Wnt ligands, in the TNBC cell line MDA-MB-231, hindered primary tumor formation in mammary fat pads and compromised lung metastasis [193]. Another Wnt antagonist, WIF1, was also found reduced in 60% of breast cancers [194], due to hypermethylation of its promoter [190,195].

Modulation of receptor activation could be a causal factor for hyperactive Wnt/β-catenin signaling in breast cancer. An aberrant splicing of *LRP5*, which removes the coding region of LRP5 that interacts with the extracellular antagonist DKK1, has been reported in human breast cancer [196]. Several studies have reported tumor suppressive effects following interference with the receptor and its association with Wnt ligands. Expression of FZD7 Wnt receptor is a characteristic of TNBC [197]. As a proof of principle, knockdown of FZD7 in the TNBC cell lines, MDA-MD-231 and BT-20, could reduce the nuclear accumulation of β-catenin, inhibit transcriptional activity of TCF7, and reduce TNBC tumorigenic abilities in vitro and in recipient mice [33]. The source of Wnt ligand in these studies could arise from the cancer cells, as autocrine Wnt signaling has been reported in a panel of breast cancer cell lines including MDA-MD-231 cells [198,199]. A pharmacological approach has also been explored to inhibit the ligand-receptor interaction. Injection of peptides containing the CRD region of FZD8 fused with Fc region of IgG (FZD8CRD-hFc) could inhibit tumor formation in the MMTV-Wnt1 model [200].

Upregulation of Dvl, a cytosolic positive regulator of Wnt/β-catenin signaling, has been found in primary breast cancers [201,202]. Moreover, genes encoding several inhibitors of Dvl were found to be epigenetically silenced in breast cancer. The naked cuticle homolog 2 (NKD2) antagonizes Wnt/β-catenin signaling by interacting with Dvl [203,204]. *NKD2* promoter methylation, associated with a reduction of NKD2 expression and activation of Wnt/β-catenin signaling, has been observed in more than 50% of human primary breast cancer samples [205]. DACT1 (Dapper/Frodo) is another antagonist of Wnt/β-catenin signaling acting through Dvl. DACT1 is frequently silenced in breast cancer cell lines, and its protein levels were noticeably reduced in breast tumors [206]. The above studies demonstrated that Wnt signaling antagonists could function as tumor suppressors, and which are frequently downregulated in breast cancer.

In the nucleus, the expression of BCL9, a co-factor that binds β-catenin and Pygo, has been found significantly increased in basal-like subtypes of breast cancer. Elevated BCL9 could modulate Wnt signaling by enhancing β-catenin targeted transcription [207]. In addition, the transcription factor LBH was found to be a direct transcriptional target of Wnt/β-catenin signaling in breast cancer cells [25,26]. LBH was found aberrantly overexpressed in MMTV-Wnt1 mammary tumors and in human basal breast cancers that display Wnt/β-catenin hyperactivation. Overexpression studies indicate that LBH suppresses mammary epithelial cell differentiation, an effect that could contribute to Wnt-induced tumorigenesis [26]. Moreover, SOX9, which is a Wnt-target in intestinal crypts [208], is also highly expressed in basal-like breast cancers. SOX9 enhanced *TCF4* transcription and Wnt/β-catenin signaling in breast cancer [209]. These studies highlight the possibility of using Wnt/β-catenin target genes as potential new markers for therapeutically challenging basal-like breast cancers.

## 5. Wnt Signaling and Breast Cancer Stem Cells

Normal tissue stem cells with active mechanisms for self-renewal were postulated to be obvious candidates for accumulating genetic mutations, initiating a malignant cell population and constituting highly tumorigenic subpopulation of cancer cells. This is the basis of the original concept of cancer stem cells (CSC) (reviewed in [210]). The CSC hypothesis has now evolved to be more complex than a simple model, with CSCs situated on top of the hierarchy. CSCs include non-stem cells that have acquire tumorigenic properties through de-differentiation either as a result of genetic mutation or in a microenvironment-dependent manner (reviewed in [211]). The term CSC is used interchangeably with tumor-initiating cells (T-IC). Numerous studies indicate that Wnt signaling contributes to cancer progression through the maintenance of CSC or T-IC. Studies of leukemia have provided the most extensive evidence for the significance of Wnt signaling in CSCs [212,213,214].

Wnt signaling also supports the action of malignant mammary stem cells in mouse models. The stem cell-enriched basal population was expanded in MMTV-Wnt1 mammary tumors, but not when MMTV was used to drive the expression of *Hras*, *Erbb2* (also known as *Neu* and *Her2*), or Polyoma middle T antigen (*PyMT*) [104,215], consistent with the idea that Wnt signaling activity is essential for stem cell maintenance. Various CSC populations have been described in different mammary tumor models. Using the MMTV-Wnt1 model, Cho et al. reported that the thymocyte antigen (Thy1)^+^, CD24^+^ population is enriched for CSCs. The Thy1^+^, CD24^+^ population makes up 1%–4% of total tumor cells and is endowed with increased tumorigenic capacity [216]. Zhang et al. demonstrated that CSCs can be identified by Wnt pathway reporter top-GFP^+^ in p53 null tumors [217]. A more recent study suggested that ΔNp63 governs tumor-initiating activity in MMTV-Wnt1 and MMTV-Myc tumors, through a Wnt signaling dependent mechanism involving Fzd7 expression [21]. Loss of ΔNp63 reduced expansion of the MaSC-enriched basal population in pre-neoplastic mammary hyperplasia, as well as led to a reduction of the Thy1+ CSC population in MMTV-Wnt1 tumors [21]. Of note, ΔNp63 has also been implicated in CSC regulation in MMTV-ErbB2 tumors, which are classified with human luminal type carcinomas, through regulation of Sonic Hedgehog (Hh) signaling pathway [218].

The first identification of CSCs in human breast cancer was achieved over ten years ago. Al-Hajj et al. demonstrated that human breast cancer cells grown as xenografts contain a subset of CD24^−^, CD44^+^ cells with increased tumorigenic capacity when compared to the remaining population. The CSC subset could be serially passaged and xenografts that were generated were histologically heterogeneous, resembling the parent tumor from which they were derived [219]. Additionally, aldehyde dehydrogenase (ALDH) was identified as a potential marker for human breast CSCs [220]. Current evidence suggests that they are two distinct CSC populations, as CD24^−^, CD44^+^ cells represent a quiescent mesenchymal-like population, whereas ALDH^+^ cells are a cycling, epithelial-like population [221]. One caveat of these studies is that variability among different breast cancer subtypes is likely extensive, and it may thus be challenging and inaccurate to designate a single CSC subset for all breast cancers. Future studies should investigate CSCs with greater resolution in each distinct breast cancer subtype, thereby providing a foundation for revealing the connection of CSCs with Wnt signaling.

## 6. Concluding Remarks

In summary, controlled activation of the Wnt signaling cascade is fundamental in embryonic organogenesis and homeostatic maintenance of the mammary gland, while its deviations associate strongly with breast tumorigenesis, especially the aggressive basal-like subtype. Significant advances have been made in the past decade towards defining the heterogeneity of mammary cells. The markers of different epithelial subpopulations and stem cells are continually being refined. With the discovery of new markers to better identify MaSCs, clear insights have been gained regarding the role of different population of MaSCs and lineage-committed stem/progenitor cells during development. The hierarchical relationships between the various recently identified populations have not been fully delineated. It remains elusive how Wnt and other signals are coordinated in vivo to determine the different populations. Thus our understanding of the role of MaSC and lineage-committed stem/progenitor cells in breast tumorigenesis remains obscure. Further studies are needed to address these questions, and understanding these questions will go a long way towards comprehending the underlying etiology of breast cancers.

## Figures and Tables

**Table 1:** Wnt target genes in the mammary gland.

Gene Name	Species	Reference
*Lef1 (Lymphoid enhancer binding factor 1)*	Mouse, Human	[19,20]
*Fzd7 (Frizzled class receptor 7)*	Mouse, Human	[21]
*Axin2*	Mouse, Human	[20,22,23,24]
*Ccnd1 (Cyclin D1)*	Mouse, Human	[20,23]
*c-Myc (Myelocytomatosis oncogene)*	Mouse, Human	[20,23]
*Dkk1(Dickkopf Wnt signaling pathway inhibitor 1)*	Mouse, Human	[20]
*LBH (Limb bud and head)*	Mouse, Human	[25,26]
*Lgr5 (Leucine rich repeat containing G protein coupled receptor 5)*	Mouse, Human	[20]
*Tgif1 (TGF* β*-induced factor homeobox 1)*	Mouse, Human	[20]
*Cox-2 (Cyclooxygenase-2)*	Mouse	[27]
*EphrinB1*	Mouse	[28]
*Msln (Mesothelin)*	Mouse	[29]
*Postn (Periostin)*	Mouse	[30]
*Procr (Protein C Receptor)*	Mouse	[18]
*Stromelysin-1 (Mmp3, Matrix metallopeptidase3)*	Mouse	[29]
*Wisp1 (Wnt-1 induced secreted protein 1)*	Mouse	[31]
*Wrch1 (Rhou, ras homolog family member U)*	Mouse	[32]
*VIM (VIMENTIN)*	Human	[33]
*TWIST*	Human	[34,35]
*SLUG (SNAI2, snail family zinc finger 2)*	Human	[34]
*WISP2 (Wnt-1 induced secreted protein 2)*	Human	[36,37]
*STRA6 (Stimulated by retinoic acid gene 6)*	Human	[38]

* Mouse nomenclature is shown when the same Wnt target gene is reported in both mouse and human.
